# Iron-Regulated Reactive Oxygen Species Production and Programmed Cell Death in Chronic Obstructive Pulmonary Disease

**DOI:** 10.3390/antiox10101569

**Published:** 2021-10-01

**Authors:** Kenji Mizumura, Yasuhiro Gon

**Affiliations:** Division of Respiratory Medicine, Department of Internal Medicine, Nihon University School of Medicine, Tokyo 173-8610, Japan; gon.yasuhiro@nihon-u.ac.jp

**Keywords:** chronic obstructive pulmonary disease, iron, reactive oxygen species, mitochondrial damage, programmed cell death, necroptosis, ferroptosis

## Abstract

Chronic obstructive pulmonary disease (COPD) is characterized by persistent respiratory symptoms and airflow limitation. However, the pathogenesis of COPD remains unclear. Currently, it is known to involve the loss of alveolar surface area (emphysema) and airway inflammation (bronchitis), primarily due to exposure to cigarette smoke (CS). CS causes epithelial cell death, resulting in pulmonary emphysema. Moreover, CS induces iron accumulation in the mitochondria and cytosol, resulting in programmed cell death. Although apoptosis has long been investigated as the sole form of programmed cell death in COPD, accumulating evidence indicates that a regulated form of necrosis, called necroptosis, and a unique iron-dependent form of non-apoptotic cell death, called ferroptosis, is implicated in the pathogenesis of COPD. Iron metabolism plays a key role in producing reactive oxygen species (ROS), including mitochondrial ROS and lipid peroxidation end-products, and activating both necroptosis and ferroptosis. This review outlines recent studies exploring CS-mediated iron metabolism and ROS production, along with the regulation of programmed cell death in COPD. Elucidating the mechanisms of these pathways may provide novel therapeutic targets for COPD.

## 1. Introduction

Chronic obstructive pulmonary disease (COPD) is a large economic and social burden as one of the top three causes of death throughout the world [1]. Chronic airflow limitation is due to a combination of small airway diseases and pulmonary emphysema, usually caused by prolonged exposure to noxious particles or gases. Cigarette smoke (CS) is the most commonly identified risk factor for COPD, with smokers having a higher COPD mortality rate than non-smokers [2]. CS contains more than 4000 identified ingredients and causes increased oxidative stress [3]. However, the precise molecular mechanisms underlying the CS-induced progressive deterioration of lung function are yet to be elucidated.

Mitochondria are the distinguishing feature of eukaryotic cells. Best known for their critical role in energy production via oxidative phosphorylation (OXPHOS), mitochondria are essential for regulating critical cellular processes such as cell death and inflammation and are hotspots of reactive oxygen species (ROS) production [4,5]. OXPHOS consists of an electron transport chain (ETC) which relies on electron transfer and a proton gradient to drive ATP production. Mitochondrial ROS (mtROS) are generated during OXPHOS and act as second messengers under physiological conditions [6,7]. The predominant mechanism of mtROS production by ETC involves the premature leakage of electrons from complexes I, II, and III, resulting in the one-electron reduction in oxygen to superoxide [8]. The formation of mitochondrial iron–sulfur clusters is essential for the generation of ETC complexes I and II. Therefore, mitochondria are closely associated with iron metabolism. Although mtROS are a natural by-product of this process, excess mtROS production induces the oxidative damage of the mitochondrial proteins, membranes, and DNA, thereby impairing the ability of mitochondria to synthesize ATP, resulting in the development of disease [9].

Outer mitochondrial membrane permeabilization and mitochondrial permeability transition are involved in apoptosis and necroptosis [10,11]. Previously, apoptosis was considered the only form of programmed cell death, whereas necrosis was recognized as unregulated cell death caused by extreme physical or chemical stress. However, recent evidence has uncovered the existence of a regulated form of necrosis, termed necroptosis [12]. Necroptosis is commonly recognized as necrotic cell death that is dependent on receptor-interacting protein kinase 3 (RIPK3) [13,14,15]. The activation of RIPK3 necessitates the formation of a RIPK1- and RIPK3-containing amyloid-like signaling complex, commonly known as the necrosome, wherein RIPK1 and RIPK3 first undergo a series of trans-phosphorylation or auto-phosphorylation events [16,17,18]. Oligomerization and intramolecular auto-phosphorylation of RIPK3 lead to the recruitment and phosphorylation of mixed-lineage kinase domain-like protein (MLKL), resulting in the formation of MLKL oligomers [16,17,19]. MLKL oligomers translocate to the plasma membrane, where they bind to specific phosphatidylinositol phosphate species and, thus, trigger plasma membrane permeabilization [20,21,22]. Apoptosis, a non-inflammatory programmed cell death pathway, does not lead to the release of damage-associated molecular patterns (DAMPs), unlike necroptosis, which induces inflammatory responses through the release of DAMPs [23]. Therefore, necroptosis, rather than apoptosis, is likely to be involved in the pathogenesis of COPD, an inflammatory disease of the airways.

In this review, we evaluate the emerging evidence favoring the contribution of CS-induced ROS production and programmed cell death to COPD pathogenesis. Mitochondria are no longer thought of as simple energy factories. Instead, they are now believed to function as central organelles to regulate cell death and inflammation. A better understanding of the effects of these processes on disease pathogenesis may reveal novel therapeutic targets for COPD.

## 2. Cigarette Smoke and ROS Production in the Lungs

Several studies have demonstrated that the oxidant burden and, consequently, the expression of oxidative stress markers is increased in the airspaces, exhaled breath, blood, and urine of smokers and patients with COPD [24]. CS, the primary risk factor for COPD, contributes significantly to the oxidant burden in the lungs. Furthermore, CS exposure activates immune cells (e.g., macrophages, neutrophils), which induce ROS production and systemic inflammation, subsequently promoting the onset and progression of comorbid cardiovascular diseases (CVD) [25]. A single puff of CS is estimated to contain an excess of 1 × 10^15^ oxidant molecules [26]. Mainstream CS comprises 92% gas-phase smoke and 8% particulate matter (or tar) [3,26]. The gas-phase of CS mainly contains ROS with short half-lives, such as superoxide radicals and nitrogen oxide, which immediately react to form the highly reactive peroxynitrite [27]. Oxidants in CS have been reported to induce excess production of mtROS in lung epithelial cells [28]. Excess mtROS can alter the expression of biomolecules, activation of signaling pathways, and function of antioxidant molecules; several mtROS have been implicated in COPD pathogenesis [29]. mtROS regulate the expression of nuclear factor erythroid 2-related factor (Nrf2), a transcription factor that regulates the expression of genes encoding proteins protecting against oxidative stress [30]. The Nrf2-antioxidant response is one of the most important mechanisms involved in COPD pathogenesis. Aged smokers and patients with COPD exhibit reduced Nrf2 expression in their pulmonary macrophages [31]. Genetic ablation of Nrf2 enhances susceptibility to CS-induced emphysema [32]. Heme oxygenase-1 (HO-1) is the inducible isoform of heme oxygenase and one of the NRF2-inducible antioxidant enzyme genes that plays a central role in defense against oxidative and inflammatory assaults in the lung [30]. CS induces HO-1 expression via activation of Nrf2 in bronchial epithelial cells [33]. Increased HO-1 expression in macrophages in the alveolar spaces of smokers has also been reported [34]. Interestingly, HO-1 siRNA augmented ROS production induced by CS in bronchial epithelial cells [35]. Adenoviral overexpression of HO-1 in lungs attenuated elastase-induced pulmonary emphysema in mice [36]. Furthermore, patients with COPD have reduced levels of HO-1 in alveolar macrophages [37] and polymorphisms of the HO-1 promoter associated with reduced HO-1 expression have been linked with increased susceptibility to emphysema [38]. These data suggest that HO-1 has cytoprotective potential against CS-induced oxidative stress in COPD. 

Nicotine is mainly contained within the particulate (tar) phase of CS and is used to help smokers quit smoking. When smoking tobacco, nicotine rapidly reaches peak levels in the bloodstream and stimulates both the sympathetic and parasympathetic ganglionic cells [39]. While nicotine addiction begins with high-affinity binding of nicotine to nicotinic acetylcholine receptors (nAChRs) in the brain [40], nAChR expression in the human airway is reported to be correlated with lung function [41]. Nicotine induced apoptosis and senescence in the bronchial epithelial cell through ROS-mediated autophagy impairment [42]. Indeed, nicotine could induce mtROS production in the human alveolar epithelial cell line A549 [43]. In contrast, Chernyavsky et al. revealed that nicotine inhibited the mitochondrial permeability transition pore opening and abolished cytochrome c release in human bronchial epithelial cells, indicating the pro-survival effect of nicotine by inhibition of mitochondria-driven apoptosis [44]. Therefore, nicotine could directly affect the bronchial epithelial cells; however, the precise role of nicotine in the pathogenesis of emphysema remains obscure. 

**Figure 1:**
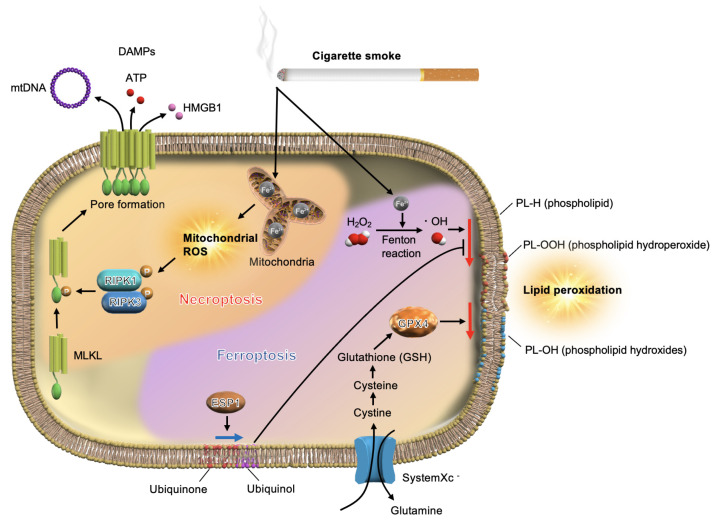
Cigarette smoke (CS)–iron axis regulates necroptosis and ferroptosis in pulmonary epithelial cells.

Iron metabolism has recently attracted increased attention as a connecting link between CS and mtROS production (Figure 1). Tobacco contains 440–1150 mg iron/g, and approximately 0.1% of this iron enters mainstream smoke [45]. Some constituents of CS release iron from ferritin, potentiating oxidative stress in lung cells [46]. Moreover, iron accumulates in the lungs of cigarette smokers [47]. CS-exposed mice showed higher non-heme iron in inflated lung sections and whole-lung homogenates than room air-exposed mice [48]. Iron-responsive element-binding protein 2 (IRP2) has been identified as an important COPD susceptibility gene, and IRP2 protein levels are increased in the lungs of individuals with COPD [49,50]. A relatively high concentration of iron can significantly increase the mitochondrial ROS levels in cells [51]. In an established mouse COPD model, mitochondrial iron chelation alleviated CS-induced impairment of airway mucociliary clearance, pulmonary inflammation, and lung injury [48]. These data suggest that iron-mediated mtROS production plays a critical role in the pathogenesis of COPD.

Along with mtROS, CS has been reported to cause lipid peroxidation [52]. The levels of F-2-isoprostanes, which are the circulating products of lipid peroxidation, are increased in smokers [52]. Lipid peroxidation plays an important role in signal transduction that initiates the inflammatory response in the lungs [53]. There is increasing evidence that aldehydes, generated endogenously during lipid peroxidation, are involved in many pathophysiological events associated with oxidative stress in cells and tissues [54]. In this sense, lipid peroxidation has been recently reported to play a critical role in the pathogenesis of COPD [55]. Moreover, iron mediates CS-induced lipid peroxidation in pulmonary epithelial cells [55], which results in pulmonary epithelial cell death by ferroptosis, a unique iron-dependent form of non-apoptotic cell death [56]. 

CS causes the accumulation of ferrous iron (Fe^2+^) in the mitochondria and cytosol. Ferrous iron overload in the mitochondria leads to mitochondrial dysfunction and mitochondrial ROS production in pulmonary epithelial cells, resulting in the activation of necroptosis. In the necrosome, RIP3 phosphorylates MLKL, and translocation of phosphorylated MLKL to the cell membrane causes direct pore formation and the release of DAMPs. The reaction of ferrous iron with hydrogen peroxide (H_2_O_2_) in the cytosol generates hydroxyl radicals (^•^OH; Fenton reaction). In “lipid peroxidation”, the hydroxyl radicals generated through the Fenton reaction oxidize the phospholipids (PL-H) and form phospholipid hydroperoxides (PL-OOH). Glutathione peroxidase 4 (GPX4) can repair peroxidation-induced lipid damage. Ferroptosis is a form of cell death driven by the loss of GPX4 and FSP1 activity and subsequent accumulation of lipid-based ROS, particularly ferrous iron-associated PL-OOH.

## 3. Programmed Cell Death in COPD

COPD is characterized by persistent respiratory symptoms and airflow limitations. Chronic airflow limitation in COPD is caused by emphysema and bronchitis associated with mucus obstruction of the airways, usually due to CS [57]. As pulmonary emphysema involves the loss of alveolar walls as a result of CS-induced epithelial cell death, apoptosis has commonly been recognized as a major cellular process leading to emphysema [58]. However, although COPD is characterized by lung inflammation that persists even after cessation of smoking [59], apoptosis occurs in the absence of inflammation owing to the limited or no release of DAMPs [60]. Therefore, the additional mechanisms have been proposed to fill in the gap between apoptosis and airway inflammation [58].

Previously, necrosis was defined as unregulated cell death caused by extreme physical or chemical stress. However, accumulating evidence has revealed the existence of a genetically programmed and regulated form of necrosis, termed necroptosis [12]. Unlike apoptosis, necroptosis directly triggers inflammation by inducing a significant release of DAMPs from the dying cells [61]. We have recently reported that CS-induced mitochondrial damage regulates necroptosis, which contributes to the pathogenesis of COPD (Figure 1) [62]. CS extract (CSE) significantly induces mtROS production and mitochondrial depolarization in pulmonary epithelial cells. CSE-induced pulmonary epithelial cell death was effectively reduced upon treatment with necrox-5, an antioxidant necrosis inhibitor, and ciclosporin A, which protects against the loss of mitochondrial membrane potential [62]. In an emphysema model, the percentage of abnormal mitochondria in the lung tissue significantly increased after three months of exposure to CS (compared with exposure to room air). Moreover, upregulated RIPK3 expression near emphysematous regions was observed in the lungs of mice exposed to CS for three months [62]. Notably, higher RIPK3 expression was found in the epithelial cells of patients with COPD than that in the epithelial cells of control subjects [62]. More recently, it has been reported that the expression of total MLKL protein in epithelium and macrophages, and phosphorylated RIPK3 and phosphorylated MLKL in the lung tissue were increased in patients with severe COPD compared to those in non-smokers or smoker non-COPD controls [63]. These findings in human clinical samples indicate that our experimental data associating mitochondrial dysfunction with necroptosis are correlated to COPD. Recently, we revealed that sphingolipids mediate CS-induced necroptosis in pulmonary epithelial cells [64]. Palmitoyl-ceramide (C16-Cer) increased in CS-exposed cells, and inhibition of ceramide-generating acid sphingomyelinase reduced CS-induced necroptosis in pulmonary epithelial cells, indicating that C16-Cer accumulation contributes to CS-induced necroptosis [64]. The release of DAMPs in response to CS-induced necroptosis triggers airway inflammation in mice [65]. These findings suggest that mitochondrial integrity and necroptosis may serve as promising therapeutic targets for COPD.

A unique iron-dependent form of non-apoptotic cell death characterized by lipid peroxidation has been discovered and termed ferroptosis [56,66]. Accumulated ferrous iron (Fe^2+^) in the cytosol reacts with hydrogen peroxide (H_2_O_2_), resulting in the generation of hydroxyl radicals (^•^OH). This is known as the Fenton reaction (Figure 1). The generated hydroxyl radicals oxidize phospholipids (PL-H) and yield phospholipid hydroperoxide (PL-OOH) in a reaction called lipid peroxidation. If lipid damage continues, ferroptosis progresses toward cell death. Glutathione-dependent lipid hydroperoxidase glutathione peroxidase 4 (GPX4) is known to prevent ferroptosis by converting PL-OOH into non-toxic lipid alcohols and phospholipid hydroxides (PL-OH) [67,68]. Recently, Doll et al. [69] and Bersuker et al. [70] reported that ferroptosis suppressor protein 1 (FSP1) protects human cells from ferroptosis. FSP1 replenishes a reduced form of ubiquinone, called ubiquinol, which protects against lipid peroxidation, a process that drives ferroptosis (Figure 1) [70,71]. Although CS-induced iron accumulation in the mitochondria induced mtROS production [48,62], a recent study revealed that CS also increases the levels of intracellular free iron and induces lipid peroxidation, resulting in ferroptosis [55]. In CS-exposed *GPx4+/−* mice, remarkably high levels of lipid peroxidation, non-apoptotic cell death, DAMP release, and enhanced COPD phenotypes of airspace enlargement and small airway thickness were observed (relative to wild type mice), all of which are attenuated in GPx4 overexpressing mice [55]. These observations, along with the findings of our previous studies, indicate that iron metabolism regulates CS-induced necroptosis and ferroptosis, both of which are implicated in the pathogenesis of COPD.

## 4. Conclusions

Current evidence demonstrates that oxidative stress regulates the new forms of cell death, namely necroptosis and ferroptosis, during COPD pathogenesis. Interestingly, iron metabolism is involved in both necroptosis and ferroptosis. The relative contributions of necroptosis, ferroptosis, and apoptosis to the pathogenesis of COPD are interesting aspects to consider. Careful considerations and further kinetic analyses of necroptosis, ferroptosis, and apoptosis to determine the temporal sequence of the molecular and cellular events using tissues recovered from a CS exposure model are required to develop valid therapeutic interventions.
